# Stem Cell Therapy and Breast Cancer Treatment: Review of Stem Cell Research and Potential Therapeutic Impact Against Cardiotoxicities Due to Breast Cancer Treatment

**DOI:** 10.3389/fonc.2014.00299

**Published:** 2014-11-03

**Authors:** Thomas E. Sharp, Jon C. George

**Affiliations:** ^1^Cardiovascular Research Center, Temple University School of Medicine, Philadelphia, PA, USA; ^2^Division of Cardiovascular Medicine, Temple University Hospital, Philadelphia, PA, USA

**Keywords:** chemotherapy–cardiotoxicity, stem cells, cardiac regeneration, differentiation, paracrine factors

## Abstract

A new problem has emerged with the ever-increasing number of breast cancer survivors. While early screening and advances in treatment have allowed these patients to overcome their cancer, these treatments often have adverse cardiovascular side effects that can produce abnormal cardiovascular function. Chemotherapeutic and radiation therapy have both been linked to cardiotoxicity; these therapeutics can cause a loss of cardiac muscle and deterioration of vascular structure that can eventually lead to heart failure (HF). This cardiomyocyte toxicity can leave the breast cancer survivor with a probable diagnosis of dilated or restrictive cardiomyopathy (DCM or RCM). While current HF standard of care can alleviate symptoms, other than heart transplantation, there is no therapy that replaces cardiac myocytes that are killed during cancer therapies. There is a need to develop novel therapeutics that can either prevent or reverse the cardiac injury caused by cancer therapeutics. These new therapeutics should promote the regeneration of lost or deteriorating myocardium. Over the last several decades, the therapeutic potential of cell-based therapy has been investigated for HF patients. In this review, we discuss the progress of pre-clinical and clinical stem cell research for the diseased heart and discuss the possibility of utilizing these novel therapies to combat cardiotoxicity observed in breast cancer survivors.

## Introduction

Advances in cancer treatments have led to a significant reduction in the incidence of mortality amongst breast cancer patients; a major accomplishment of today’s cancer therapies. The 5-year survival rate for females in the United States is 89%, and 78% at 15 years (1). Associated with increased breast cancer survival is an increase in cardiovascular co-morbidities (2). The scope of this issue has not been adequately studied and is not readily ascertained from clinical trial data on emerging chemotherapeutic agents. Clinical trials often consist of small cohorts of patients with under representation of specific patient populations and exclude those with co-morbidities. In addition, the incidence of adverse cardiac events has usually not been evaluated. It is not surprising that novel cancer therapeutics can cause adverse cardiac events given the fact that cancer drugs influence cell survival (3–5). In concert with these novel reagents, some cancer treatment plans incorporate classical chemotherapeutics (anthracyclines) that are known to be more toxic to the cardiovascular system (6–8). Whether the pathways (survival and growth) by which these agents inhibit tumor progression overlap with those which preserve cardiovascular cell physiology, remains largely unknown. In our view, there is a need to investigate different therapeutics strategies to combat any adverse cardiovascular event observed in cancer patients.

Cancer therapeutics cause cardiomyopathy in large part by causing the death of cardiac myocytes and supportive tissue (4, 5, 9–14). Therefore, cell therapies that repair existing myocardium or regenerate new myocardium to replace lost tissue could improve cardiac function in cancer survivors. Researchers and physician-scientist have been investigating cell-based therapy since the early 1980s (15). In striving to understand the basic biology of adult stem cells, tremendous progress has been made in comprehending their therapeutic potential against disease states like acute myocardial infarction (AMI) and ischemic heart disease, culminating with numerous clinical trials since early 2002 (16). While still somewhat controversial, the scientific community is beginning to define the mechanism(s) responsible for the beneficial effects of those stem cell therapies tested to date. Regardless of the treatment strategy used to prevent or reverse adverse cardiovascular events in breast cancer patients, it will become increasingly important to screen patients, optimize treatment strategies, and monitor cardiac function prior to-, during-, and after cancer treatment.

## Breast Cancer and the Etiology of Cardiotoxicity

In 2013, the projected number of new *in situ* and invasive breast cancer cases was just shy of 300,000 (1). Breast cancer death rates have been dropping since the early 1990s (1), due to better awareness by women to have annual mammograms, which has led to earlier detection and better success of treatment strategies. With over 2.9 million women living in the United States with a medical history indicating breast cancer (17), there has become a greater need for an understanding of the therapeutics utilized to combat breast cancer and their potential effects on other organ systems.

Physicians have a variety of treatment options and strategies to slow, inhibit, and/or eliminate breast cancer. Newer generation chemotherapeutics have the capability of targeting specific pathways; usually interrupting cell survival (3, 8, 11, 18–20), growth (21), and proliferation (3, 8, 11, 18, 22, 23). Selective targeting therapeutics are a true testament to the amount a basic and clinical research that has gone into comprehending cancer biology over the last several decades. Ideal cancer therapeutics should affect cancer cells without effects on normal tissues. Unfortunately even target specific agents have “off target” effects on normal cells in the heart and other tissues. Radiation therapy has also been improved as a therapeutic against breast cancer. With advances in technology, clinicians have the ability to more accurately direct the radiation treatment while minimizing the dose need; but still there are major side effects observed with both treatment options, and the incidence of cardiotoxicity is on the rise (24).

While treatment may lead directly to cardiovascular dysfunction in some patients, in others it may hinder their ability to cope with preexisting or newly acquired cardiovascular diseases such as ischemic heart disease and hypertension. It is important to point out that only a fraction of patients in chemotherapeutic clinical trials have reported adverse cardiac events (25, 26); 4–7% of patients in initial trials suffered from cardiotoxicity when treated with monoclonal antibody chemotherapeutics, which manifested itself as a decrease in left ventricular ejection fraction (LVEF) (27). This percentage was drastically increased (27%) when patients were treated concurrently with adjuvant chemotherapeutics, like anthracyclines (14).

There are several hypotheses as to mechanism by which chemotherapeutic treatment initiates and/or exacerbates cardiotoxicity observed in breast cancer patients (4, 11, 12). The more classical drugs, like anthracyclines, most notably Doxorubicin, have been linked to greater increase in reactive oxygen species (ROS) causing more stress at the cellular level (10, 28, 29). In cardiomyocytes, there is an abundance of mitochondria, which produce free radicals from anthracyclines, which are taken-up by the cell (30). This predisposes cardiac tissue to create high levels of ROS. This suggests high levels of newly formed ROS limits the amount of antioxidants that are found endogenously. With depletion of these much needed antioxidants, homeostasis is not maintained leading to an unfavorable cellular environment. A single basic research study, by De Angelis et al., looked directly at mechanisms by which chemotherapeutics are cardiotoxic and their effects on endogenous cardiac stem cells (CSC’s) (31), which are thought to be involved in endogenous cardiac repair. It was shown that classic chemotherapeutics (anthracyclines) increased ROS formation, caused DNA damage, induced p53 expression and cell cycle arrest in the G2/M phase, while decreasing CSC growth (31).

Cardiotoxicity due to radiation therapy predominantly leads to pericardial and coronary vasculature damage. While early radiological practices lead to constrictive pericarditis; new technology and techniques to minimize the exposure of the heart to radiation and the incidence of pericarditis is still largely unknown due to limiting number of years post-technology development (32). Cell types, which are part of the coronary vascular framework have been shown to induce inflammation and lead to cardiovascular events, which can cause ischemic heart disease (33). In a study which compared the effects of left- or right-sided radiation demonstrated an increase in coronary stenosis in patients who received left-side treatment; specifically the left anterior descending coronary artery (9). Again, with new techniques and better technology being utilized, this adverse event can be minimized.

More reviews have come forth over the last several years discussing chemotherapeutic cardiotoxicity (3, 4, 8, 19, 20, 34–38) and there has been the formation of guidelines with clinical interdisciplinary cross talk between oncologists and cardiologists (11, 39–41) to more effectively treat the toxicity to organs such as the heart. Again, whether the primary treatment strategy is pharmacological or radiological, physicians have come to a consensus that adjuvant therapy increases the probability of initiating or exacerbating cardiotoxicity in breast cancer patients (4, 11, 12, 14). New basic, translational, and clinical studies will be essential to define the mechanisms of cardiotoxicity of chemotherapeutics and radiation therapy. It will also be important to carefully follow the increasing number of breast cancer survivors, to define their long-term cardiovascular risk.

## Stem Cell Therapy

In this review, we suggest that stem cell therapy should be considered for cancer survivors who develop cardiomyopathy. Currently, one of the most impressive aspects of stem cell therapy for the heart is the wide variety of cell types that could be considered as potential candidates through pre-clinical (Table 1) and clinical research (Table 2). This reflects the true unmet need for a therapeutic avenue to be developed in order to treat and prevent the progression and manifestation of heart failure (HF) in patients who suffer cardiac injuries, like myocardial infarction or breast cancer therapy-induced cardiomyopathy. Here, we discuss endogenous cardiac regeneration and some of the more popular cell types that are being looked at as potential candidates for cell-based therapy.

**Table 1:** **Overview of animal studies with stem cell therapy**.

Study	Host	Etiology of dysfunction	Route of administration	Outcomes
**BONE-MARROW MONONUCLEAR CELLS (BMMNCs)**				
Orlic et al. (42)	Mice	Ligation of LAD	IM	↑LV function
				Trans-differentiation
Mathieu et al. (43)	Dog	Ligation of LAD	IM	↑LV function, ↓Scar
				↓Brain natriuretic protein
				Neovascularization
Bel et al. (44)	Sheep	Ligation of CX	IM	No Δ LVEF or remodeling
Waksman et al. (45)	Pig	Permanent occlusion	IM	↓Scar
				Trans-differentiation
				Angiogenesis
**BONE-MARROW-DERIVED HEMATOPOIETIC STEM CELLS (HSCs)**				
Balsam et al. (46)	Mice	Ligation of LAD	IM	No trans-differentiation
Kajstura et al. (47)	Mice	Ligation of LAD	IM	↑LV function, ↓Scar
				Trans-differentiation
**MESENCHYMAL STEM CELLS (MSCs)**				
Hatzistergos et al. (48)	Pig	I/R	IM	↑LV function, ↓Scar
				Trans-differentiation
				Homing of endogenous SCs
Cai et al. (49)	Rat	Ligation LAD	IM	↑LV function
				↓Remodeling
Quevedo et al. (50)	Pig	I/R	IM	↑LV function, ↓Scar
				Trans-differentiation
				Angiogenesis
Schuleri et al. (51)	Pig	I/R	IM	↑LV function, ↓Scar
				Angiogenesis
**CARDIAC STEM CELLS (CSCs)**				
Linke et al. (52)	Dog	Occlusion of LAD	IM	↑LV function
				Trans-differentiation
				Angiogenesis
Beltrami et al. (53)	Rat	Ligation of LAD	IM	↑LV function
				↓Remodeling
				Trans-differentiation
Fischer et al. (54)	Mice	Ligation of LAD	IM	↑LV function
				↓Scar
				Trans-differentiation
				Angiogenesis
Li et al. (55)	Mice	I/R	IC	↑LV Function
				↓Remodeling
				Trans-differentiation

*↑, Increase; ↓, decrease; No Δ indicates change; CX, circumflex coronary artery; LAD, left anterior descending coronary artery; I/R, ischemia-reperfusion; LV, left ventricle; IM, intramyocardial; IC, intracoronary*.

**Table 2:** **Overview of clinical trials with stem cell therapy**.

Study	No. patients	Route of administration	Primary end-point	Outcomes
**BONE-MARROW MONONUCLEAR CELLS (BMMNCs)**				
Perin et al. (56)	Cell = 14	IM	Echocardiography	↑LV function
	Control = 7			↓Remodeling
				↓NYHA Class
Perin et al. (57)	Cell = 11	IM	Echocardiography	No Δ LV function
	Control = 9			↑Exercise capacity
				↑Perfusion
Galinanes et al. (58)	Cell = 14	IM (during CABG)	Dobutamine stress	↑LV function
	No Control		Echocardiography	↑Wall motion
Hendrikx et al. (59)	Cell = 10	IM (during CABG)	MRI	No Δ LV function
	Control = 10			↓Remodeling
				↓NYHA class
Fischer-Rasokat et al. (42) (TOPCARE-DCM)	Cell = 33	IC	MRI	↑LV function
	No Control		LV angiography	↑Wall Motion
**BONE-MARROW-DERIVED HEMATOPOIETIC STEM CELLS (HSCs)**				
Vrtovec et al. (60)	Cell = 28	IC	Echocardiography	↑LV function
	Control = 27	
Vrtovec et al. (56)	Cell = 55	IC	Echocardiography	↑LV function
	Control = 55	
Patel et al. (61)	Cell = 10	IM (during CABG)	Echocardiography	↑LV function
	Control = 10	
**MESENCHYMAL STEM CELLS (MSCs)**				
Hare et al. (62) (POSEIDON)	Cell = 31	IM	Computed tomography	No Δ LV function
	No Control			↓LVEDV
				↑Physical performance
Karantalis et al. (63)	Cell = 6	IM (during CABG)	MRI	↑LV function, ↓Scar
	No control	
**CARDIAC STEM CELLS (CSCs)**				
Bolli et al. (64) (SCIPIO)	Cell = 16	IC	Echocardiography	↑LV function, ↓Scar
	Control = 7		MRI
Makkar et al. (65) (CADUCEUS)	Cell = 17	IC	MRI	No Δ LV function, ↓Scar
	Control = 8			

*↑, increase; ↓, decrease; No Δ, no change; Cell, Cell-treated patients; CABG, coronary artery bypass graft surgery; LVEDV, left ventricular end-diastolic volume; NYHA, New York Heart Failure Association; LV, left ventricle; IM, intramyocardial; IC, intracoronary*.

### Cardiac regeneration

The heart has a limited capacity for repair after injury. This limited repair capacity is the bases for cardiac dysfunction after ischemic insult or damage from cancer chemotherapeutics. Why the heart has such a limited ability to repair itself and how cell therapy might enhance repair is an important topic in need of further study. Most questions about cardiac regeneration are still not resolved. Interestingly, fish and other less developed species have an ability to regenerate lost portions of their hearts, primarily via proliferation of surviving myocytes that reenter the cell cycle (66, 67) post insult. This characteristic is also present in the fetal and early neonatal mammalian heart, but is generally absent in adult mammalian human heart tissue. Regardless of the robustness of endogenous cardiac repair it is clear that the adult human heart cannot repair itself after multiple forms of injury which can lead to HF.

Adult cardiac myocytes are largely withdrawn from the cell cycle. Therefore the loss of myocytes with disease requires new myocyte formation to prevent cardiac functional decline. New myocytes could be derived from old myocytes that reenter the cell cycle or from a stem cell population with cardiogenic capacity. Some laboratories have demonstrated there is a small rate of turnover in myocytes in the adult heart (68–70) but not at a sufficient rate to repair the heart back to basal functional levels post injury. Other than cardiac transplantation, there is no therapy, which ultimately addresses the issues caused by myocardial injury and the progression of cardiac remodeling. With chemotherapeutic agents and radiation therapy affecting survival, growth, and proliferation pathways, while increasing oxidative stress and DNA damage, frank loss of heart muscle, and deterioration of myocardial support structure mimics other types of cardiac injury such as myocardial infarction. Whether this cardiotoxicity occurs acutely or chronically in breast cancer patients is unclear but the end result is most notably DCM or RCM (20, 31, 71–73).

The fundamental principle that the human heart does not have an adequate endogenous repair mechanism has led to the discovery of isolating adult stem cells for use as a therapeutic for treating and preventing HF, which has exploded in the scientific research community and has given a new sense of hope to the idea of cell-mediated repair of the heart.

### Bone-marrow-derived stem cells

The bone-marrow is a diverse tissue that houses many cell types, including a variety of stem cells (56, 60, 74–76). Due to the ease of acquisition, with already approved clinical methods and their relatively high abundance, bone-marrow-derived stem cells have been and continue to be investigated as a possible source of cells that can be applied toward cardiac regeneration. This cell source is one of the most widely examined in pre-clinical experimentation and clinical trials to date. Here, we outline the major populations and their potential as cell therapy.

#### Unfractionated bone-marrow mononuclear cells

Bone-marrow mononuclear cells are a heterogeneous mixture of multiple cell types [hematopoietic stem cells (HSCs), mesenchymal stem cells (MSCs), endothelial progenitors, and other more committed cell population] (57–59, 74). Through a density gradient centrifugation, bone-marrow mononuclear cells (BMMNCs) are isolated easily from whole bone-marrow fraction. With the easy of isolation and low maintenance *in vitro*, these cells have been utilized as a source of cell therapy in many animal models. In the acute MI setting, BMMNCs have shown much promise (42, 43). In contrast under chronic conditions of HF, the jury is still out; conflicting results in large animal models (43–45) and smaller scale preliminary clinical trials (77–79) still leave many questions as to the true mechanism(s) of action and the efficacy of this cell population. In a pig (45) model of HF, transplantation of BMMNCs provided no therapeutic benefit in terms of left ventricular (LV) function, but the study described an increase in angiogenesis and reduced infarct size. In another large animal study post infarct (43), BMMNC therapy showed an improvement in LV function, and reduced probrain natriuretic peptides (BNP) levels in the plasma, will also sparking angiogenesis.

In the clinical arena, the results have been similar to the observations in the basic research community. The first clinical evaluation of BMMNCs as a therapeutic was performed by Perin et al. (77); 21 patients were enrolled (14 cell-treated and 7 control). Functional improvements were observed at 2–4 months; in patients receiving cell therapy there was a 9% increase in LVEF as compared to baseline and a reduction in the end-systolic volume (77). Subsequent other trials confirmed these observations of improved cardiac function with intramyocardial injection of BMMNCs (78). In contrast, when cell were injected directly in the core of the damage region in 20 patients all beneficial effects were negated, there was no significant difference in LVEF or wall thickness by MRI (80). These vastly different outcomes have many factors, which may be playing a role in the results obtained, particularly the location of the injected cells. The microenvironment plays a pivotal role in the efficacy and any potential benefit cell therapy may have, as observed in these contrasting clinical trials (one with injection into the border zone of the infarct and the other into the core). In studies, which investigated the role of BMMNC therapy for non-ischemic cardiomyopathies there were promising results (81). BMMNCs therapy increase the regional LV function and improved microvascular function in Transplantation of Progenitor Cells and Recovery of LV Function In Patients With Non-ischemic Dilative Cardiomyopathy (TOPCARE-DCM), which enrolled 33 patients to receive intracoronary administration of BMMNCs (81).

Studies of BMMNCs as a viable option for cell therapy have yielded inconsistent results both at the bench and in small scaled clinical trials, this is largely due to the heterogeneity of the cell population and the yield of actual progenitors in each isolation for therapeutic use. Larger scale trial’s must be run in order truly understand what effect(s) this cell type may be having as an option for cardiac regenerative therapy.

#### Hematopoietic stem cells

Hematopoietic stem cells reside within the bone-marrow and commit to two different cell lineages, myeloid and lymphoid. The major cell surface marker which is used to distinguish this sub-population of cells from other progenitors which reside is in the bone-marrow is cluster differentiation 34 (CD34) (82–84); a transmembrane cell adhesion protein that has implicated in the literature to denote stem cells, which has a hematopoietic or vascular lineage. HSCs are mobilized from the bone-marrow into the peripheral blood during ischemic events to begin the process, which leads to revascularization (75). Researchers and clinicians felt that by isolating this population of cells and reintroducing them in more concentrated numbers would promote greater revascularization than observed by endogenous mechanisms post cardiac injury (46, 47, 75).

Numerous clinical trials have been performed evaluating CD34+ cells in patients with both ischemic (61) and non-ischemic (56, 60) cardiomyopathy. Vrtovec et al. (56) looked to understand the beneficial effects of this cell population against non-ischemic cardiomyopathy by delivering the cells intracoronary to 55 of the 110 patients enrolled; this led to a ~5% increase in LVEF, improvement in the 6-min walk test and decreased probrain natriuretic peptide plasma levels. A 5-year follow-up study was able to demonstrate that the transplantation of these cells had an effect over a sustained period much longer than most trials (60). The true mechanism by which this population of cells is having an effect is still not understood, but the major consensus amongst those in the field would be an increase in perfusion via revascularization. Preliminary clinical work with CD34+ hematopoietic cells is promising for both ischemic and non-ischemic cardiomyopathy, as with most of the cell types discussed here, a major limitation is the small sample sizes in these trials and lack of understanding as to the mechanism of action, which is due to an inability to apply standard methods utilized in basic research, toward human patients (i.e., immunohistochemistry, fluorescent microscopy, and molecular analysis).

An important issue concerning this cell population is the fact that only autologous transplantations have been performed. For the average patient who has been enrolled in such Clinical trials to date, this resident population of cells can be easily harvested and utilized for cell-based therapy. In terms of the subset of patients discussed here, this may not be the case. For individuals who have received or continue to undergo chemotherapy and radiation treatment, the CD34+ HSC population may be exhausted or non-existent all together (85, 86). This would subsequently eliminate this population of progenitors as a viable option for cell-based therapy to treat any cardiomyopathy induced by chemotherapeutic treatment of breast cancer. If this population of stem cells were to be beneficial against cardiotoxicity, it may be necessary for patients to undergo isolation prior to cancer treatment, so that cells could be isolated and expanded for future autologous cell-based therapy if needed. Other populations within the bone-marrow do exist and do not have to be autologous in nature for transplantation.

#### Mesenchymal stem cells

Bone-marrow-derived MSCs are a sub-population of cells characterized by their adherence in culture (87). They also have begun to characterize a host of cell surface marker, which identifies this population within isolated bone-marrow. The majority of MSCs express CD29, CD73, CD90, and CD105 while being negative for hematopoietic lineage markers CD34 and CD45 (87, 88). Others have demonstrated sub-populations within the MSCs, which express these markers and a plethora of others (89, 90). The multipotentiality of these cells to differentiate into osteoblast, chondrocytes, adipocytes *in vitro* (91–94) is well documented and cardiomyocytes *in vivo* (95–97), which is still controversial (98). Paracrine signaling is one of the major mechanisms thought to elicit improvement by MSC therapy (48, 99) in the heart. This is due to release of numerous growth- (48), anti-apoptotic- (100, 101), and/or angiogenic- (49, 102) factors helping protect the myocardium and augment some of the adverse remodeling. Furthermore, MSCs demonstrate a capacity to engraft in a large animal model of MI (50, 51, 103) and have shown an ability to evade immune rejection (52, 104–106). In recent studies, results indicate MSC contributed directly to inhibition of inflammatory responses (107, 108), which may be the mechanism behind the observed reduction in scar size in both animal models and clinical trials (51, 62, 63). While there is still skepticism, this characteristic could allow MSCs to be used as an allogeneic source of cells, overcoming the need for isolation and expansion of autologous cell sources.

With many clinical trials looking to understand the beneficial effect of numerous different cell types in patient suffering from cardiac related dysfunction, MSCs in recent years has become more popular for translational applications in patients (62, 63). Hare et al. (62) investigated MSC’s and their effect(s) on 15 of the 30 patient enrolled in the clinical trial Percutaneous Stem Cell Injection Delivery Effects on Neomyogenesis (POSEIDON). This trial look to see if there was any dose dependent effect of MSC’s in patients who were suffering from ischemic cardiomyopathy (ICM). The data demonstrated, at all three doses, that MSC administration was favorable when measuring end-points of quality of life, functional capacity and ventricular remodeling (62). Krantalis et al. (63), in the Prospective Randomized Study of Mesenchymal Stem Cells Therapy in Patients Undergoing Cardiac Surgery (PROMETHEUS) trial, investigated the injection of MSC’s in six patients receiving coronary artery bypass graft surgery (CABG). Those regions of the myocardium, which received cell therapy demonstrated a decrease in scar mass compared with baseline at 18 months follow-up (63). An overwhelming number of clinical trials that are “recruiting” encompass MSC’s therapy exclusively or as part of their treatment strategy (109). At this point, MSC’s are becoming more promising for clinical applications and widely investigated for the utility of cardiac regeneration in the clinical setting.

### Cardiac stem cells

Cardiac-derived stem cells have also been in the spot light of animal investigations and recently, clinical trials (53, 54, 65, 110–113). The discovery that the heart is in fact an organ, which has the ability to have cellular turnover and renewal (both of myocytes and non-myocytes) refutes the long withstanding dogma that the heart is a post-mitotic organ. This renewal is thought to be derived from a population of stem cells, which reside as niches within the myocardium (110). New methodology has been developed over the last decade to isolate (53) and characterize these cells *in vitro* (53, 111) and investigate their therapeutic potential. The isolation of CSCs has given hope that these cells will be predisposed to an increased probability of neomyogenesis as compared to other cell types discussed previously.

#### C-kit (+)/hematopoietic lineage (−) CSCs

This cell population was first described in 2003 by Beltrami et al., cells were isolated from a rodent heart (53). The manuscript describes a cell population isolated from cardiac tissue that expressed a tyrosine kinase receptor c-kit, now a known marker of stemness (53). This population not only fit the classical definition of a “stem cell” (self-renewing, clonogenic, and multipotent) but also differentiated into cardiomyocytes, smooth muscle cells, and endothelial cells *in vitro* and *in vivo* (53, 110, 111). Human cardiac c-kit+ positive cells were isolated some 4 years later (111). Since then, injection of isolated c-kit+ CSCs and studying the beneficial effects has been overwhelming; multiple laboratories and basic research studies have demonstrated that post injection an alleviation of LV dysfunction and adverse remodeling, while showing the elicit response of regeneration due to injection (54, 55, 114). With such positive outcomes in rodent models (54, 55, 115), this cell type was soon moved to a pre-clinical large animal model. Bolli et al. (64) investigate the role on intracoronary infusion of CSCs 3 months post-MI and found a significant difference in LVEF as compared to vehicle treated animals, while demonstrating increased wall thickness and beneficial changes in the maximal developed pressure, as well as, a lower diastolic pressure. With that, this work in the large animal model laid the ground work for a human clinical trial investigating the efficacy and safety of CSC’s in patients. The Stem Cell Infusion in Patients with CardiOmyopathy (SCIPIO) clinical trial update discussed the infusion into the coronary circulation, 1 million c-kit+/lineage – CSC’s into 16 patients with LV dysfunction (113). The authors concluded that these cells produced better LV systolic function through reduction of scare size in patients with MI, and further clinical trials should be performed (113). With promising results in the phase I trial, CSCs are bidding to become the superior choice in choosing a cell type for cardiac cell therapy. While clinical trials are ongoing, there has only been one small animal study investigating the therapeutic potential of CSC therapy post chemotherapeutic cardiotoxicity, this study as discussed above (cardiotoxicity section) looked to solidify the mechanism by which the cardiotoxicity occurs and utilized c-kit+ CSCs as a therapeutic intervention to combat the adverse effects observed (31). De Angelis et al. (31) concluded that cell-based therapy promoted regenerative capacity of the myocardium, improved cardiac pump function, and decreased mortality.

Collectively, with all the successes of pre-clinical and clinical trials to date, there is much more work that is needed to fully understand the therapeutic potential of cell-based therapy for all types of cardiac disease states regardless of the etiology.

## Cell Therapy Potential for Chemotherapeutic/Radiation-Induced Cardiotoxicity in Breast Cancer Patients

**Figure 1:**
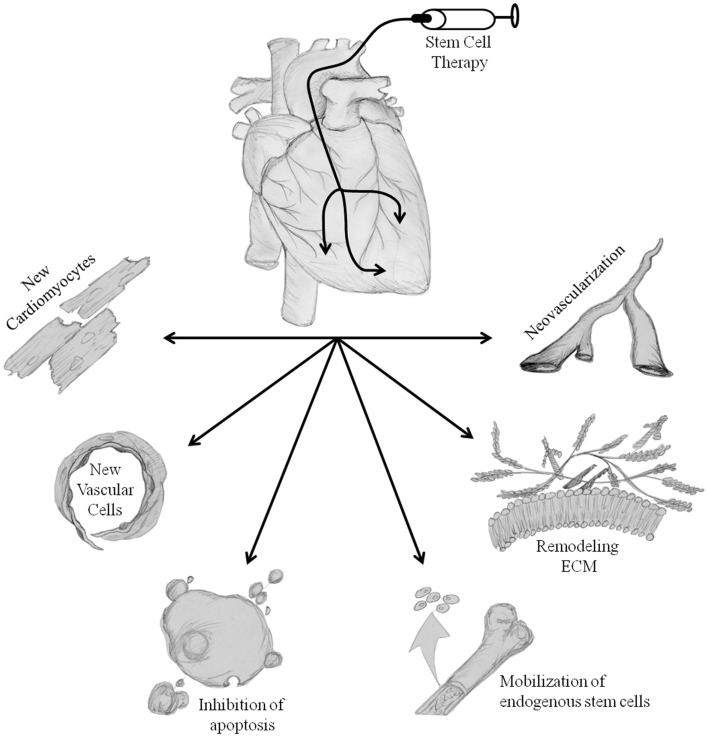
**Proposed mechanisms of stem cell-mediated repair**. Transplantation of stem cells into the heart initiates repair of damaged tissue. The hypothesized repair mechanisms are both direct and indirect, trans-differentiation of stem cells into new cardiomyocytes and vascular cells, inhibition of apoptosis, mobilization of endogenous cell populations, alterations in ECM remodeling, and neovascularization. Collectively, these processes reduce adverse cardiac remodeling, increase the possibility of perfusion, repair/regenerate damaged tissues, and ultimately improve left ventricular cardiac pump function & patients clinical end-points. Illustration credit: Thomas E. Sharp III.

With the plethora of basic science and clinical research performed on isolating and characterizing a number of adult stem cells to be utilized for cardiac cell therapy in the past two decades, we as a field still do not know which cell type, and/or combination of cells will be most beneficial. The work has yielded some rewards despite most questions still not having answers; we now understand that multiple tissues have population of stem cells that have the capacity to be beneficial toward heart function post injury and inhibit adverse remodeling, while improving quality of life in patients suffering from many different cardiac disease states (15, 16, 42, 50, 51, 56, 62, 64, 69, 83, 100, 102, 113). Despite not fully understanding the mechanism of action, the field has a general consensus on ways in which stem cell therapy is working to improve cardiac function (Figure 1); animal studies have shown beneficial effects of stem cell therapy through paracrine factor secretion (48, 99, 100), trans-differentiation into multiple cell types, which help to improve cardiac function (92, 116) and through homing of endogenous stem cells to the site of injury (48, 76). The cell types discussed above do not all work with the same mechanism of action; it has been demonstrated that MSCs most likely work through paracrine factor production and secretion (48, 51, 74, 117–119), while BMMNCs and CSCs have the ability to form new blood vessels for better perfusion (46, 47, 53–55, 59, 75–77, 79, 85, 86, 89, 96, 110, 120, 121) and create new myocyte from transplanted cells (53, 55, 64, 111–115, 122, 123). Below, we discuss the major mechanisms and how they may be beneficial toward patients suffering from cancer treatment-related cardiotoxicity.

### Trans-differentiation of transplanted cells

The logical explanation for using stem cell therapy to repair the heart is the idea in which transplanted cells will form new myocardium replacing lost or damage tissue. As obvious as this may seem, data acquired thus far in the field of cardiac regeneration would suggest that little trans-differentiation is actually occurring, and that this is probably the least likely mechanism of action for the observed improvements post therapy. Much of the debate still goes on as to the amount or proportion of beneficial effects that should be attributed toward trans-differentiation. Still highly controversial is the notion that cell populations derived from the bone-marrow (HSC’s, MSC’s, and CD34+ SC’s) form new cardiac myocytes; numerous laboratories have evidence supporting such notions (124, 125), while others contest these conclusions (46, 126). Alternatively, some suggest that the mechanism of action is fusion of the injected cells with endogenous surviving myocytes (127, 128). Discussed in more detail below, most would agree that the major mechanism of action may be paracrine factor production and secretion (100, 101). While in the acute MI disease model, there is strong evidence for trans-differentiation (53, 110, 129–131); in the post-MI HF large animal model the data would suggest that the amount of trans-differentiation observed is insufficient to explain the significant increase in cardiac function post injury and after therapeutic intervention (64). In recent years, the debate has turned more toward understanding the proportion of new myocyte formation in the different cell types (discussed above) and how the quantification of this trans-differentiation is proportionate or disproportionate to the improved cardiac function. In patients suffering cancer therapy cardiotoxicity, trans-differentiation of transplanted stem cells may allow for the replacement of cells that may otherwise have died from necrosis (132) or other proposed mechanisms (3, 6, 14, 18–20, 31, 36, 38, 133) due to chemotherapeutic treatment and in turn limit the amount of fibrosis which develops. In limiting the fibrosis, in patients suffering from chemotherapeutic/radiation cardiotoxicity, we would anticipate less adverse remodeling and subsequently better outcomes over time. As discussed above, this mechanism is likely unable to account for any or all the benefit which may occur in these patients post-stem cell treatment.

### Neovascularization

The creation of new blood vessels *de novo* may be of great benefit to patients who suffer from chronic or persistent coronary occlusion, which develops into ICM. This may occur in cancer patients due to the anti-angiogenic nature of classical chemotherapeutics (3, 5, 40) and frank loss of vascular structure from radiation therapy. On the contrary, those who suffer from non-ischemic cardiomyopathy, it is difficult to see the beneficial aspects of utilizing cells which have demonstrated in experimental models to create new vasculature. What may be the most important mechanism or alternative action, which has allowed for the most benefit, is paracrine factor production/secretion and signaling.

### Paracrine signaling

In reality, the inability (up to now) to solidify the mechanism of action by which stem cells act on the heart has led to great emphasis on the paracrine hypothesis (100). This concept hypothesizes that transplanted cells modulate the myocardial milieu in the injury site by secreting factor that signal to the surrounding cells and tissue(s). Paracrine signal may in fact promote a multitude of reparative and regenerative processes, like: promoting cell survival, the inhibition of cell apoptosis, promoting a new blood vessel formation, favorable changes to the extracellular matrix (ECM), modulation of the inflammatory response which occurs upon injury, and activation/homing of endogenous stem cell populations to the site of injury. This signaling can also play a key role in the ability for transplanted stem cells to thrive in a harsh environment by autocrine signaling and positive feedback loops. In concert, these actions promote better LV function and slower progression of remodeling and development of HF.

#### Cell survival and inhibition of apoptosis

Numerous basic research studies have suggested the production and secretion of paracrine factors [like, insulin like growth factor-1 (IGF1) and secreted frizzled-related protein-2 (SFRP2)] inhibit cardiomyocyte apoptosis (101, 134). Another parameter, which may assist in the pro-survival hypothesis is the modulatory affect of the stem cells toward the immune response (101, 108, 135). In augmenting the immune response one could hypostulate less activation of the positive feedback loop within the innate and adaptive immune responses to cardiac injury. This in turn, would limit cell death and deposition of ECM proteins, which could potential preserve the myocardium and LV function.

#### Angiogenesis

In a recent study of a rodent model of MI, Duran et al. (136) was able to demonstrate the production of specific paracrine factors by stem cells, which promote angiogenesis and incorporation of stem cells into newly formed vasculature *in vivo*. Multiple cell populations have been described as producing angiogenic factor such as: fibroblast growth factor-2 and -7 (FGF) (137), platelet-derived growth factor (PDGF) (138), and vascular endothelial growth factor (VEGF) (100, 137). With chemotherapeutics being highly toxic and anti-angiogenic (3, 5, 40), utilizing stem cell therapy to maintain/repair vasculature and promote the neovascularization of areas, which may be lacking blood supply is an important idea. While some may caution the notion of promoting neovascularization and angiogenesis in patient suffering from cancer in fear of potentially promoting vascularization of present tumors and causing metastasis, one should withhold their reservations, as techniques, which are used to deliver the stem cells are usually performed locally within the organ [intracoronary delivery (55, 64, 65, 102, 121, 139, 140) and intramyocardial injection (77, 119, 141–143)]. Aside from this minor concern, this therapeutic benefit from stem cell administration is one of the more promising for patients who have been administered chemotherapeutics or undergone radiation treatment, which are hailed for the ability to inhibit vasculature formation.

#### ECM remodeling

Under the paracrine hypothesis, stem cells have been ascribed the ability to augment deleterious alterations in the ECM (138, 144–146). Post stem cell therapy has shown in rodent models of MI to reduction in scar size, reduced fibrosis, and subsequently inhibition of LV remodeling (74, 118, 137, 140, 146–148). While there is no significant scar formation in patients who suffer direct cardiotoxicity from chemotherapy, the reduction in fibrosis may play an important role in these patients. In having the capacity to change the cell niche with which myocardial cells reside is an important factor, as most chemotherapeutic cardiotoxicity is not due to ischemia, rather a change in the abundance of fibrosis in the cellular milieu (3, 5, 13, 20, 40) and cell death.

#### Homing of endogenous progenitor populations

With a wide variety of paracrine factors being produced by stem cells, specific factors have been implicated in mobilizing and homing endogenous stem cells pools to the site of injury or sites of transplantation of exogenous cells (48, 140). Such factor include: stem cell-derived factor (SDF) (138), hepatocyte growth factor (HGF), and IGF (100, 101). These factor collectively permit endogenous stem cell homing, proliferation, and differentiation into myocardial cell types (myocytes and vascular cells), concurrently with some of the other beneficial effects observed with such factors as IGF [which has demonstrated to be pro-survival (101)]. In patients who have undergone chemotherapy, this mechanism of mobilizing native stem cells is probably not likely, as with most of the basic research studies performed thus far have concluded that chemotherapeutics are deleterious to endogenous stem cell population (23, 31, 132, 149).

#### Autocrine signaling

While the paracrine signaling hypothesis discusses the therapeutic nature of growth factor signaling on endogenous tissue(s), the hypothesis has also given rise to scientific investigation of this signaling on the cells, which produces them. Many laboratories have demonstrated that autocrine signaling of growth factors and factors of stemness are necessary for self-renewal, maintenance, survival, and growth. FGF (150–152) has been shown to drive self-renewal, inhibit cellular senescences, and inhibit apoptosis. While others have demonstrated that SDF plays a critical role in survival and maintenance of the stem cell(s) (153). This paracrine/autocrine signaling may help enhance the other effects that transplanted cells may have on endogenous tissue by allowing the transplanted cells to be retained and produce more of these factors, while also enhancing the possibility of trans-differentiation, due to longer retention.

While these major mechanisms of action are being vetted in animal models, one thing has become certain; the therapeutic benefit of stem cells is not exclusively made up of a single mechanism but more likely multi-factorial and in different proportions depending on the stem cell population chosen for therapeutic intervention. While most studies have not looked at stem cells therapy for chemotherapeutic/radiation cardiotoxicity, some basic research publications have indicated improvement with stem cell administration (31).

## Challenges Facing Cell-Based Therapy

With any novel therapeutic in the R & D phase there are many unknowns and obstacles, which must be investigated. Clinical trials of stem cells therapy for patients suffering from cardiac pathologies similar to those observed in patients with chemotherapeutic/radiation cardiotoxicity have shown promise (56, 62, 65, 77, 78, 113, 121, 154, 155), but there is more work needed to be done in order to truly understand the mechanisms behind the improved cardiac function. Once recognizing and establishing more concrete comprehension of the therapeutic benefit of such an intervention, the medical community will be able to make a more informed decision as to whether or not stem cells are a viable option for treatment of chemotherapeutic cardiotoxicity. There are many questions, which are still unresolved, for example: (1) understanding what stem cell populations are optimal for regeneration, (2) is there a dose dependent effect, and (3) what time points should cell therapy be administered and how frequent. These issues can only be answered with more careful planned pre-clinical and clinical trials, not only for more broad cardiac disease states (like acute MI and congestive HF), but also in concentrating on understanding the negative effects of chemotherapeutic/radiation cardiotoxicity and the potential of cell-based therapy in this context. With this, we believe that stem cell-based therapy is one of the frontiers still left in medicine today. There is an enormous amount of potential for regenerative medicine in context of the heart and will probably be a viable option for the treatment of chemotherapeutic/radiation-induced cardiotoxicity.

## Conflict of Interest Statement

The authors declare that the research was conducted in the absence of any commercial or financial relationships that could be construed as a potential conflict of interest.
